# The Therapeutic Effects of *Crocus sativus L*. and Its Constituents on the Improving Treatment of Azoospermia: A Narrative Review

**DOI:** 10.1002/hsr2.73129

**Published:** 2026-08-26

**Authors:** Faraz Khakshour, Melika Hadad Tehran, Jamal Jalili Shahri, Farid Zeynali, Fahimeh Lavi Arab

**Affiliations:** ^1^ Department of Laboratory Sciences, School of Paramedical and Rehabilitation Sciences University of Medical Sciences Mashhad Iran; ^2^ Immunology Research Center Mashhad University of Medical Sciences Mashhad Iran; ^3^ Vascular and Endovascular Research Center Mashhad University of Medical Sciences Mashhad Iran; ^4^ Department of Urology, Faculty of Medicine Mashhad University of Medical Sciences Mashhad Iran; ^5^ Department of Immunology, Faculty of Medicine Mashhad University of Medical Sciences Mashhad Iran

**Keywords:** antioxidant, azoospermia, *Crocus sativus L.*, infertility, male, oxidative stress

## Abstract

**Background and Aims:**

Azoospermia, defined as the complete absence of sperm in the ejaculate, is a major cause of male infertility and is classified into obstructive and non‐obstructive forms. While obstructive azoospermia is primarily mechanical in origin, non‐obstructive azoospermia (NOA) is characterized by impaired spermatogenesis and is frequently associated with oxidative stress, inflammation, and hormonal dysregulation. *Crocus sativus L*. and its bioactive constituents exhibit antioxidant and anti‐inflammatory properties that may target underlying pathophysiological mechanisms involved in NOA. This review discusses the potential adjunctive role of *Crocus sativus L*. in the context of NOA, with emphasis on oxidative stress–related pathways.

**Methods:**

This paper is a narrative review identified through relevant literature of electronic databases, including PubMed, Scopus, Web of Science, and Google Scholar. Peer‐reviewed experimental studies written in English that examined the antioxidant and anti‐inflammatory effects of *Crocus sativus L*. and its bioactive compounds on male reproductive health.

**Results:**

Available studies suggest that *Crocus sativus L*. bioactives may help attenuate oxidative stress, modulate inflammatory mediators, and support testicular structure and spermatogenesis in experimental models of chemical, radiation, and metabolic injury. Evidence indicates improvements in antioxidant enzyme activity, reductions in lipid peroxidation, and potential preservation of hormonal balance. However, clinical data are limited, and variability in dosage, extract composition, and study design precludes definitive conclusions about reproductive outcomes.

**Conclusion:**

*Crocus sativus L*. and its bioactive compounds could have supportive effects in contexts of testicular oxidative and inflammatory stress, but their clinical relevance in NOA remains uncertain. Further well‐controlled human studies are needed to clarify their potential therapeutic role.

## 1 Introduction

**Figure 1:**
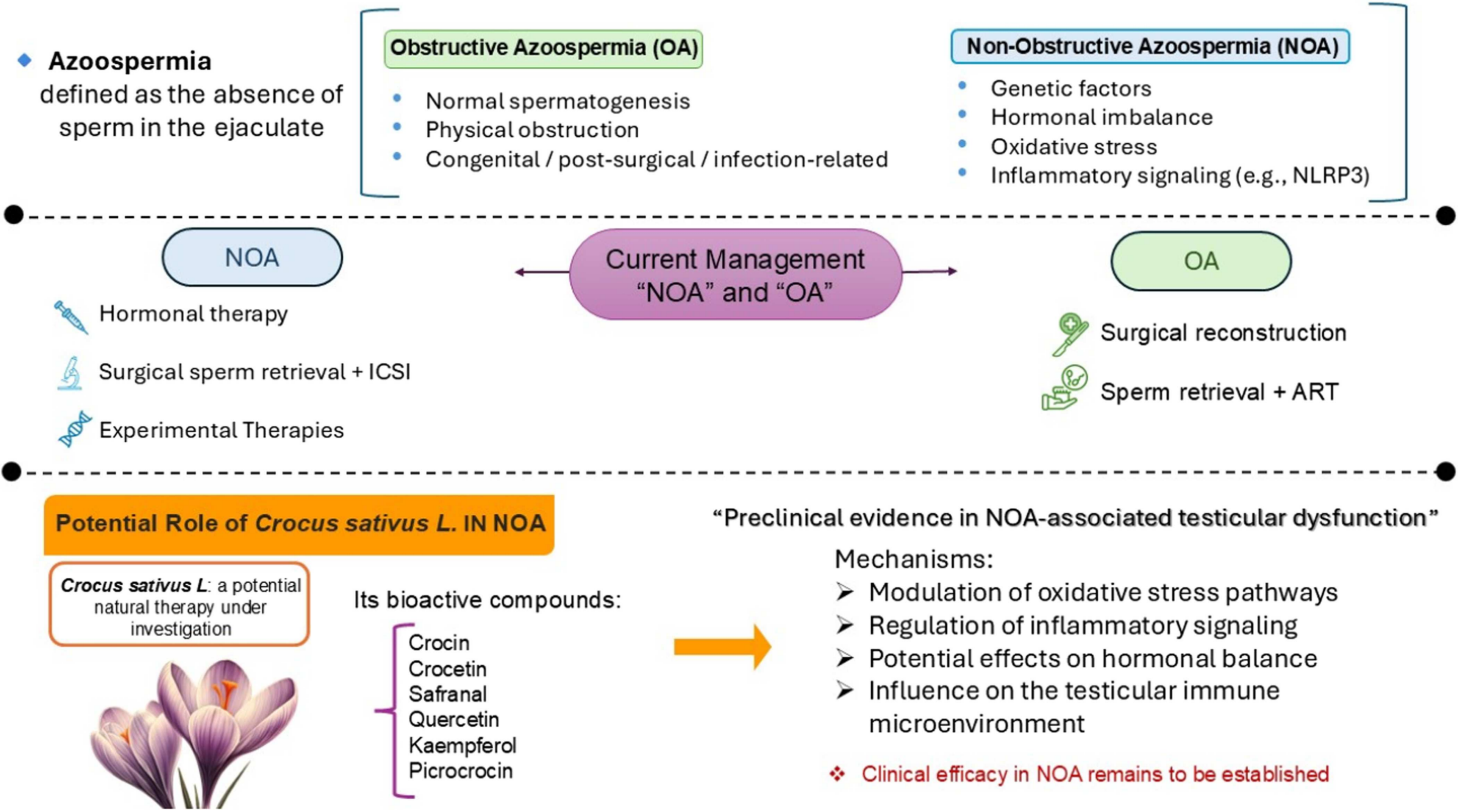
Pathogenesis and treatment of azoospermia with focus on *Crocus sativus L*. This figure summarizes the role of inflammation in azoospermia and the main current treatment options. It also highlights the potential benefits of *Crocus sativus L*. and its active compounds.

One of the main causes of male infertility is azoospermia, which affects about 1% of all males and accounts for 10%–15% of infertile men globally. Azoospermia is defined as the absence of sperm in the ejaculate [1]. It is broadly divided into two categories: NOA, which is characterized by impaired or absent germ cell development due to intrinsic testicular dysfunction, and OA, which is caused by physical blockage of the male reproductive tract despite preserved spermatogenesis [2]. Although assisted reproductive technologies and surgical sperm retrieval methods can result in fertilization in certain situations, like OA, there are still few treatment options for NOA, and results vary greatly. Assisted reproductive techniques like intracytoplasmic sperm injection (ICSI) are typically used after surgical sperm retrieval methods (such as conventional testicular sperm extraction (c‐TESE) and microdissection TESE (mTESE)) [3]. The underlying cause determines how well hormonal therapy works. In hypogonadotropic hypogonadism, gonadotropin therapy can effectively restore spermatogenesis, and withdrawal and endocrine management may help with azoospermia associated with exogenous testosterone or anabolic steroid use. Hormonal treatments, however, typically have little effect in idiopathic non‐obstructive azoospermia without reversible endocrine suppression [4, 5]. There are currently no effective medical treatments for genetic causes of male infertility, such as Y‐chromosome microdeletions or karyotypic abnormalities (like Klinefelter syndrome). The mainstay of management is ICSI in conjunction with genetic counseling and assisted reproductive technologies, such as testicular sperm retrieval when practical. Preimplantation genetic testing may be taken into consideration in certain situations to lower the risk of transmission to progeny [6] Figure 1. Although they do not explain all causes, oxidative stress and inflammation may play a role in testicular dysfunction in certain types of male infertility, especially in certain NOA cases. By harming germ cells and compromising Leydig and Sertoli cell function, excessive reactive oxygen species (ROS) disturb the testicular microenvironment and ultimately impede spermatogenesis [7]. Traditional medical systems have long used medicinal plants to treat problems related to male reproduction. Numerous plant‐derived extracts may have positive effects on the male gonads through androgenic, antioxidant, anti‐inflammatory, and spermatogenesis‐enhancing mechanisms, according to an increasing amount of experimental and clinical data [8, 9, 10]. Among these, the medicinal plant *Crocus sativus L*., which contains bioactive compounds like crocin, crocetin, safranal, and kaempferol, has shown anti‐inflammatory and antioxidant properties in a variety of experimental systems and has been studied for its ability to shield testicular tissue from oxidative stress [11, 12]. Table 1 With an emphasis on oxidative stress‐related mechanisms and their possible supportive role in azoospermia, this narrative review attempts to provide an overview of the most recent experimental and clinical data pertaining to the biological effects of *Crocus sativus L*. and its main constituents in relation to male infertility.

**Table 1:** Biological effects of *Crocus sativus L.* and its main constituents on male reproductive function.

Bioactive compounds	Molecular formula	Chemical class	Primary mechanism of action	Ref
Crocin	C44H64O24	Water‐soluble carotenoid	Reduces lipid peroxidation, restores SOD, CAT, GPx, GSH; downregulates NF‑κB/TLR‑4, Bax, Caspase‑3; activates Nrf2/AKT–FOXO	[13]
Crocetin	C20H24O4	Dicarboxylic carotenoid	Enhances oxygen transport, scavenges ROS, increases antioxidant enzymes, modulates Nrf2	[13]
Safranal	C10H14O	Monoterpene aldehyde	Potent free‑radical scavenger; stabilizes membranes; ↑GSH, GPx; ↓MDA, NF‑κB, TNF‑α, IL‑6; activates Nrf2	[13]
Picrocrocin	C16H26O7	Monoterpene glycoside	Radical‑scavenging; precursor of safranal	[13]

## 2 Methods

This study is a narrative review summarizing the current evidence on the effects of *Crocus sativus L*. on azoospermia and testicular dysfunction. A relevant literature search was conducted across PubMed, Scopus, Web of Science, and Google Scholar for studies published from 1990 to 2025. The search strategy utilized combinations of the following keywords: “azoospermia,” “non‐obstructive azoospermia,” “male infertility,” “*Crocus sativus L*.,” “crocin,” “crocetine,” “oxidative stress,” “spermatogenesis,” and “testicular inflammation.” Inclusion criteria comprised peer‐reviewed experimental (in vivo and in vitro) studies, preclinical investigations, and relevant clinical studies published in English that evaluated the effects of *Crocus sativus L*. or its bioactive constituents on male reproductive health, spermatogenesis, oxidative stress, inflammation, or azoospermia‐related outcomes. Exclusion criteria included duplicate publications, non‐English articles, and reports with insufficient methodological or outcome data. As this manuscript was designed as a narrative review, PRISMA guidelines were not formally applied. Unlike systematic reviews, narrative reviews aim to provide a broad and critical overview of the available evidence rather than a comprehensive quantitative synthesis. Nevertheless, to minimize selection bias, the literature search was conducted using multiple electronic databases and predefined inclusion and exclusion criteria.

## 3 Pathophysiology of Non‐Obstructive Azoospermia

Disruptions at the pre‐testicular and testicular levels of the male reproductive system can cause azoospermia, a complex disorder. The hypothalamic‐pituitary‐gonadal axis is frequently dysfunctional in pre‐testicular causes, which results in insufficient hormonal stimulation of spermatogenesis. In addition to environmental and toxic insults, testicular causes include intrinsic defects in germ cell development brought on by genetic abnormalities like Y‐chromosome microdeletions or chromosomal disorders like Klinefelter syndrome [14]. Together with these well‐established processes, oxidative stress and inflammation also play a role in testicular dysfunction and impaired spermatogenesis, especially in non‐obstructive azoospermia.

### 3.1 Role of Oxidative Stress

When the body's antioxidant defense mechanisms are overpowered by ROS production, oxidative stress arises, causing oxidative damage to the testicular tissue's lipids, proteins, and DNA [15]. There may be a connection between OS and significant spermatogenesis impairment because men with NOA frequently exhibit elevated systemic indicators of oxidative imbalance, such as increased lipid peroxidation and decreased total antioxidant capacity [16]. Normal sperm function, including capacitation, hyperactivation, and acrosome reaction, depends on physiologically regulated levels of ROS. However, oxidative stress, which results in lipid peroxidation, DNA damage, mitochondrial dysfunction, and germ cell apoptosis, is caused by excessive ROS production or compromised antioxidant defenses. Activated leukocytes during inflammatory conditions, immature or aberrant spermatozoa, and mitochondrial activity in germ cells are the main sources of ROS. Severe forms of male infertility, such as NOA, are caused by persistent oxidative imbalance, which also damages spermatogenesis and disturbs the testicular microenvironment [17, 18].

### 3.2 Role of Inflammation

Inflammation plays a critical role in the pathophysiology of azoospermia, particularly through disruption of the testicular microenvironment and impairment of spermatogenesis. Pro‐inflammatory cytokines such as TNF‐α, IL‐1β, and IL‐6 can interfere with the function of Sertoli and Leydig cells, leading to reduced testosterone production, impaired germ cell support, and induction of germ cell apoptosis [19]. These alterations compromise the integrity of the seminiferous epithelium and contribute to spermatogenic failure. Recent evidence highlights the involvement of inflammasomes, particularly the NLRP3 inflammasome, in sterile inflammation of the male reproductive tract. Activation of NLRP3 promotes the release of inflammatory mediators such as IL‐1β, which has been associated with impaired testicular function and defective spermatogenesis. Increased NLRP3 expression has been observed in azoospermic patients, suggesting a mechanistic link between inflammasome activation and male infertility [20]. Chronic inflammation can also disrupt the blood‐testis barrier, allowing immune cells to infiltrate the seminiferous tubules and directly damage germ cells. This immune‐mediated injury impairs germ cell development and maturation, ultimately contributing to azoospermia [21, 22]. Additionally, inflammatory conditions such as epididymo‐orchitis may lead to structural damage and fibrosis, further increasing the risk of infertility.

## 4 Current Therapeutic Approaches

The management of NOA remains clinically challenging and requires an individualized approach based on the underlying etiology. Among available interventions, mTESE has become the preferred surgical technique for sperm retrieval, with reported success rates varying depending on the underlying pathology, including genetically associated conditions such as Klinefelter syndrome [14, 23]. Varicocelectomy is indicated in infertile men with clinically significant varicocele and may improve semen parameters and reproductive outcomes, including assisted reproductive success, although its effectiveness in restoring spermatogenesis in non‐obstructive azoospermia remains limited [24]. Endocrine therapies, including human chorionic gonadotropin (hCG) and human menopausal gonadotropin (hMG), are well‐established treatments for men with hypogonadotropic hypogonadism and may effectively stimulate spermatogenesis in appropriately selected patients [5, 25, 26]. Nevertheless, therapeutic outcomes in NOA remain heterogeneous and largely depend on the underlying etiology and the extent of intrinsic testicular damage.

## 5 Therapeutic Potential of *Crocus sativus L*. in Non‐Obstructive Azoospermia

### 5.1 Antioxidant and Anti‐Inflammatory Properties

Oxidative stress plays a critical role in male infertility, with elevated levels contributing to lipid peroxidation and impairment of sperm motility and viability. While physiological levels of ROS are required for normal sperm function, excessive ROS production disrupts cellular homeostasis and induces structural and functional damage [27]. Recent studies have highlighted the antioxidant, anti‐inflammatory, and immunomodulatory properties of *Crocus sativus L*., suggesting its potential relevance in supporting male reproductive function through modulation of inflammatory mediators such as IL‐6 and TNF‐α [13, 28]. Among its bioactive constituents, crocins represent a group of carotenoid glycosides, with up to 47 distinct molecules identified, and crocin‐1 (trans‐4‐GG) being the most abundant. Crocins account for approximately 10%–15% of the dry stigma weight and are primarily responsible for the antioxidant activity of the plant. Crocetin, the aglycone metabolite of crocin, also exhibits significant antioxidant capacity [29, 30, 31, 32]. From a mechanistic perspective, crocin acts as a potent direct free radical scavenger, specifically neutralizing hydroxyl and peroxyl radicals. This action prevents oxidative damage to critical cellular components, including lipids, proteins, and DNA. Experimental evidence suggests that these antioxidant effects are dose‐dependent; for instance, crocin, which was solubilized in PBS at a concentration of 500 µM, has been shown to significantly bolster antioxidant defenses and mitigate cellular injury under induced oxidative stress [33]. However, clinical and translational evidence suggests that the antioxidant effects of crocin may be context‐dependent. Although crocin supplementation has been associated with improvements in antioxidant capacity, meta‐analytic findings indicate that crocin does not consistently produce significant reductions in malondialdehyde (MDA), a key biomarker of lipid peroxidation linked to sperm function and vitality [34, 35]. These findings highlight the importance of dosage, treatment duration, and clinical context in determining its therapeutic efficacy. Preclinical studies provide further mechanistic insights into the protective effects of Crocus sativus and its constituents. For example, administration of a lyophilized aqueous extract of Crocus sativus at 80 mg/kg has been shown to attenuate sodium valproate‐induced testicular damage in albino rats by reducing chromosomal abnormalities, DNA fragmentation, and tissue injury. Treatment also improved the mitotic index and enhanced antioxidant defenses, including catalase (CAT) and reducing antioxidant power (RAP), while lowering MDA levels [36]. Similarly, crocetin (solubilized in DMSO) at 50 μM has demonstrated radioprotective effects in the testes of pubertal mice, reducing ionizing radiation‐induced cellular damage through modulation of oxidative stress‐related markers such as PARP1, PCNA, SOD2, and HuR [37]. In addition, an aqueous solution of crocin at 12.5, 25.0, and 50.0 mg/kg has been reported to mitigate chemotherapy‐induced gonadotoxicity. In experimental models, crocin improved reproductive parameters and reduced oxidative stress markers, including MDA, following busulfan exposure [38]. Additionally, an aqueous solution of crocin (at 40 mg/kg) has demonstrated protective effects against doxorubicin‐induced testicular injury in experimental models, where it reduced seminiferous tubule atrophy, edema, and structural disorganization. These protective effects were accompanied by improvements in antioxidant defense markers, including GSH, SOD, CAT, and TAS, along with reductions in oxidative stress indicators such as MDA and TOS, suggesting a potential role in mitigating chemotherapy‐induced reproductive toxicity [39]. Collectively, these findings suggest that crocin and related compounds may help preserve testicular function under conditions of oxidative stress, although their clinical efficacy in severe forms of male infertility, such as non‐obstructive azoospermia, requires further investigation. Table 2.

**Table 2:** Summary of anti‐inflammatory and antioxidant roles of saffron in azoospermia treatment.

Compound	Study model	Concentration	Key parameters evaluated	Findings	ref
Crocin	Sodium valproate‐induced damage (Albino rats)	80 mg/kg	DNA fragmentation, Chromosomal abnormalities, CAT, RAP, MDA	Attenuated testicular damage, improved mitotic index, and enhanced antioxidant defenses while lowering MDA levels.	[36]
Crocetin	Ionizing radiation‐induced damage (Pubertal mice)	50 μM	PARP1, PCNA, SOD2, HuR	Demonstrated radioprotective effects by modulating oxidative stress‐related markers.	[37]
Crocin	Busulfan‐induced gonadotoxicity (Experimental models)	12.5, 25, 50 mg/kg	Reproductive parameters, MDA	Mitigated chemotherapy‐induced toxicity and improved overall reproductive parameters.	[38]
Crocin	Doxorubicin‐induced testicular injury (Experimental models)	40 mg/kg	GSH, SOD, CAT, TAS, MDA, TOS	Reduced seminiferous tubule atrophy and edema; improved antioxidant markers and reduced oxidative indicators.	[39]
Crocin	Oxidative stress‐induced cellular injury (In vitro)	500 μM	Free radical scavenging (hydroxyl and peroxyl)	Acted as a direct free radical scavenger, neutralizing ROS and reducing damage to lipids, proteins, and DNA.	[33]

### 5.2 Protection of Testicular Function

*Crocus sativus L*. has demonstrated significant protective effects on testicular function across various experimental models of testicular injury. These protective effects are primarily mediated through antioxidant, anti‐apoptotic, and hormonal regulatory mechanisms that help preserve spermatogenic integrity and testicular architecture. Pretreatment with Aqueous Extract of *Crocus sativus L*. at 100 mg/kg has been shown to mitigate cadmium‐induced testicular damage by improving sperm count and viability, restoring seminiferous tubule structure, and enhancing cell proliferation, as reflected in improved Johnsen scores. These effects were accompanied by reduced lipid peroxidation, highlighting its ability to attenuate oxidative stress‐induced damage [40]. Similarly, crocin (dissolved in normal saline) has demonstrated protective effects against cyclophosphamide‐induced reproductive toxicity (at 20 mg/kg) by preserving glutathione redox balance, improving sperm quality, and supporting hormonal pathways involved in spermatogenesis. In addition, crocin, as evidenced by decreased caspase‐3 activity and improved histological features, significantly reduced testicular apoptosis [41]. The antioxidant properties of *Crocus sativus L*., dissolving it in sterile distilled have also been confirmed in metabolic injury models, such as diabetes, where treatment at 200 mg/kg enhanced the activity of endogenous antioxidant enzymes, including catalase (CAT) and glutathione peroxidase (GPx), thereby reducing oxidative damage in testicular tissue [42]. Furthermore, at 50 mg/kg crocin dissolving in normal saline has shown protective effects against radiation‐induced testicular injury through modulation of the AKT/FOXO signaling pathway, resulting in reduced oxidative stress and inflammation, preservation of testosterone levels, and maintenance of testicular structure [43]. In ischemia/reperfusion injury models, crocin dissolved in normal saline improved histological parameters, restored antioxidant enzyme activity, and enhanced testosterone levels (at 100 mg/kg), suggesting its ability to mitigate oxidative and structural damage even when functional sperm parameters were not significantly improved [44]. Similarly, in acrylamide‐induced testicular toxicity, crocin was dissolved in physiological saline at 50 mg/kg, restored hormonal balance, enhanced antioxidant defenses, and improved histopathological features, including seminiferous tubule integrity and epithelial thickness [45]. Table 3.

**Table 3:** Summary of the role of saffron in maintaining testicular health.

Compound	Injury model	Dosage	Key mechanisms/markers	Findings	Ref
*Crocus sativus L*.	Cadmium‐induced damage	100 mg/kg	Lipid peroxidation, Johnsen scores	Improved sperm count/viability; restored seminiferous tubule structure and cell proliferation.	[40]
Crocin	Cyclophosphamide toxicity	20 mg/kg	Glutathione redox balance, Caspase‐3	Reduced apoptosis; improved sperm quality and preserved histological features.	[41]
*Crocus sativus L*.	Diabetic metabolic injury	200 mg/kg	CAT, GPx	Enhanced endogenous antioxidant enzymes and reduced oxidative damage in testicular tissue.	[42]
Crocin	Radiation‐induced injury	50 mg/kg	AKT/FOXO signaling	Reduced inflammation/oxidative stress; maintained testosterone levels and testicular architecture.	[43]
Crocin	Ischemia/Reperfusion (I/R)	100 mg/kg	SOD, GPx, Testosterone	Improved histological parameters and restored antioxidant enzyme activity.	[44]
Crocin	Acrylamide‐induced toxicity	50 mg/kg	Hormonal balance, Epithelial thickness	Restored LH/FSH/Testosterone levels; improved seminiferous tubule integrity.	[45]

### 5.3 Mechanisms of Action of *Crocus sativus L*.'S Bioactives on Spermatogenesis

The bioactive constituents of *Crocus sativus L*., particularly crocin, appear to influence spermatogenesis through multiple interconnected mechanisms. *Crocus sativus L*. has also been implicated in supporting hormonal balance, a crucial factor in maintaining normal spermatogenesis. Disruption of hormonal homeostasis can impair spermatogenic function. While Crocus sativus has been associated with endocrine modulation under certain pathological conditions, dose‐dependent effects have been reported. In a rat model, oral administration of saffron (dissolved in physiology serum solution) at 200 mg/kg for 28 days was associated with significant reductions in spermatogenesis index (SI), tubular differentiation index (TDI), and repopulation index (RI), potentially linked to decreased serum testosterone levels, whereas lower doses (50,100 mg/kg) did not produce similar alterations [46]. Beyond endocrine modulation, crocin has been reported to attenuate oxidative stress–mediated testicular damage induced by environmental and toxic exposures, including nicotine and electromagnetic fields. Similarly, nicotine‐exposed models demonstrated dose‐dependent protective effects of crocin dissolved in normal saline (12.5,50 mg/kg), with improvements in sperm motility, viability, and testosterone levels. Protective effects were also observed in electromagnetic field exposure models, where crocin (dissolved in distilled water) at 50 mg/kg reduced apoptosis and restored reproductive parameters [47, 48]. Furthermore, given the close interaction between psychological stress and the hypothalamic–pituitary–gonadal axis, the anxiolytic and antidepressant effects of *Crocus sativus L*. mediated through central neurotransmitter modulation may indirectly support male reproductive function. Nevertheless, these neuroendocrine effects require further validation in clinical settings [49, 50, 51]. Table 4.

**Table 4:** Mechanisms of action of bioactive compounds from *Crocus sativus L*. on spermatogenesis.

Compound	Mechanism	Experimental model	Dose	Key reproductive outcomes	Ref
Crocin	Endocrine modulation	Rat model	200 mg/kg	↓ Spermatogenesis index (SI), ↓ TDI, ↓ RI, ↓ testosterone	[46]
Crocin	Oxidative stress attenuation	Toxic exposure models (nicotine)	12.5,50 mg/kg	↑ Sperm motility, ↑ viability, ↑ testosterone	[47]
Crocin	Anti‐apoptotic effect	Electromagnetic field exposure	50 mg/kg	↓ Testicular apoptosis, restoration of reproductive parameters	[48]

## 6 Clinical Studies and Human Trials

While direct clinical trials specifically targeting NOA remain scarce, the therapeutic potential of *Crocus sativus L*. is increasingly extrapolated from its effects on severe male infertility and experimental models of spermatogenic failure. Clinical studies suggest that these compounds may alleviate symptoms of mild to moderate depression through mechanisms involving neurotransmitter regulation, anti‐inflammatory effects, and neuroprotection, with some trials reporting efficacy comparable to conventional antidepressants such as fluoxetine and a favorable safety profile [52, 53, 54]. In addition, saffron supplementation has demonstrated potential benefits in reducing anxiety symptoms [55, 56]. Because psychological stress, anxiety, and depression are increasingly recognized as factors that can negatively influence hormonal balance and spermatogenesis, their management may indirectly support male reproductive health [57]. Clinical evidence on saffron and male reproductive outcomes is limited and mixed. A large randomized, double‑blind, placebo‑controlled trial in men with idiopathic oligoasthenoteratozoospermia found no significant improvements in sperm count, motility, morphology, or seminal antioxidant capacity with 60 mg/day saffron for 26 weeks. In contrast, a smaller trial in infertile men following varicocelectomy reported improved sperm motility, but no changes in concentration or morphology, with a comparable dosing regimen. Separately, standardized saffron preparations have shown systemic antioxidant effects and improvements in sexual function in various clinical contexts, suggesting indirect but unproven relevance to male reproductive health [27, 58].

## 7 Discussion

Current research on the possible involvement of *Crocus sativus L*. and its bioactive components in azoospermia, with a focus on non‐obstructive azoospermia (NOA), is compiled in this narrative review. According to the literature currently available, saffron‐derived compounds may have an impact on a number of biological pathways, such as oxidative stress, inflammatory signaling, hormonal regulation, and the immune microenvironment, that are implicated in testicular dysfunction [13, 28, 43].

Under toxic, metabolic, or inflammatory conditions, crocin, crocetin, and related constituents have been shown in experiments to have cytoprotective and antioxidant properties that may help maintain the structure of seminiferous tubules and support spermatogenic processes [36, 39, 41].

However, there is still variation in the strength of the available evidence. Direct translation to human NOA is limited because a large portion of the data is derived from preclinical models [40, 45]. Furthermore, there is a great deal of variation among studies, especially when it comes to extract composition, dosing strategies, length of treatment, and outcome measures. This makes comparisons difficult and precludes drawing firm conclusions about the effectiveness of treatments. With some reports suggesting possible endocrine changes at higher exposures, emerging findings also imply that the biological effects of saffron bioactives may be dose‐dependent, highlighting the significance of carefully assessing safety and appropriate dosing [33, 36].

Clinically speaking, surgical sperm retrieval procedures and specific endocrine treatments remain the mainstays of NOA management, with saffron‐based approaches currently being considered experimental. The quantity and scope of human studies are still restricted, and meta‐analytic data show that decreases in lipid peroxidation indicators like MDA do not always follow increases in total antioxidant capacity [34, 35]. When taken as a whole, these findings demonstrate the difficulties in converting mechanistic antioxidant effects into reproductive outcomes that are clinically significant.

## 8 Limitations

Although this review highlights promising evidence regarding the potential role of *Crocus sativus L*. in azoospermia, several important limitations should be emphasized. First, the majority of available data derive from preclinical animal models, and extrapolation of effective dosages to humans remains challenging. For example, doses commonly used in animal experimental studies would correspond to several grams per day in an average adult male when directly scaled by body weight, raising concerns regarding feasibility, long‐term safety, and potential toxicity.

Moreover, excessive antioxidant supplementation has been suggested to disrupt physiological ROS signaling, potentially impairing spermiogenesis through reductive stress mechanisms. Thus, high‐dose antioxidant therapy may not be universally beneficial and could pose unintended reproductive risks.

In addition, variability in saffron extract preparation, bioactive compound concentration, resorption rates, and product quality may significantly influence therapeutic outcomes. Differences in cultivation conditions, processing methods, and potential contamination further complicate standardization and reproducibility across studies.

Finally, while preclinical findings are encouraging, the true therapeutic efficacy of saffron in NOA remains speculative due to the limited number of well‐powered randomized controlled trials. Therefore, rigorously designed clinical studies with standardized formulations and clearly defined dosing strategies are essential before clinical recommendations can be made.

## 9 Conclusion

Our review highlights the therapeutic potential of *Crocus sativus L*. and its active compounds in addressing azoospermia. The antioxidant and anti‐inflammatory properties of active compounds are crucial for reducing oxidative stress, modulating hormonal balance, and enhancing spermatogenesis. While existing studies demonstrate promising effects in both animal and human models, further clinical research is needed to validate these findings and establish standardized treatment protocols. Given the increasing interest in herbal medicine for reproductive health, *Crocus sativus L*. emerges as a potential natural remedy for male infertility.

## Author Contributions

**Faraz Khakshour:** writing – original draft, investigation, data curation. **Melika Hadad Tehran:** writing – original draft, investigation, data curation. **Jamal Jalili Shahri:** writing – review and editing. **Farid Zeynali:** project administration, conceptualization. **Fahimeh Lavi Arab:** supervision, writing – review and editing, conceptualization.

## Funding

The authors have nothing to report.

## Disclosure

All authors have read and approved the final version of the manuscript [Fahimeh Lavi Arab] had full access to all of the data in this study and takes complete responsibility for the integrity of the data and the accuracy of the data analysis.

## Conflicts of Interest

The authors declare no conflicts of interest.

## Transparency Statement

The [Fahimeh Lavi Arab] affirms that this manuscript is an honest, accurate, and transparent account of the study being reported; that no important aspects of the study have been omitted; and that any discrepancies from the study as planned (and, if relevant, registered) have been explained.

## Data Availability

Data sharing not applicable to this article as no datasets were generated or analyzed during the current study.
